# Supplementary material for: Unsupervised fake news detection on social media using hybrid Gaussian Mixture Model
Source: PLoS One. 2025 Aug 18;20(8):e0330421. doi: 10.1371/journal.pone.0330421 (PMC12360590; doi:10.1371/journal.pone.0330421)
Supplement: Attachment — Submitted filename: Response-to-Reviewers.docx [file pone.0330421.s002.docx]

**Original Manuscript ID:** PONE-D-24-50144

**Original Article Title: “**Unsupervised fake news detection on social media using hybrid gaussian mixture model”

**To:** PLOS ONE Editor

**Re:** Response to reviewers

Dear Editor,

Thank you for allowing a resubmission of our manuscript, with an opportunity to address the reviewers’ comments.

We are uploading (a) our point-by-point response to the comments (below) (response to reviewers, under “Response-to-Reviewers Files*”*), (b) an updated manuscript with yellow highlighting indicating changes (as “Revised Article with Changes Highlighted*”*), and (c) a clean updated manuscript without highlights (“Manuscript”*).*

Best regards,

**Reviewer 1, Concern # 1. (Please read, present, and discuss at least related work regarding the following aspects (please respond individually):- the use effect of word embeddings to encode fake news [1]- use of transformer embeddings to encode fake news [2]- how document embeddings are used to encode fake news [3]- real-time architectures for fake news and harmful content detection and mitigation [4] (e.g., ContCommRTD https://doi.org/10.1109/TKDE.2024.3417232) or [5] (e.g. StopHC https://arxiv.org/abs/2411.06138(- using social network features for fake news detection [6] (e.g., https://doi.org/10.1016/j.knosys.2024.111715)- the effect of multilingual transformers on fake news detection [7] (e.g., http://ceur-ws.org/Vol-3180/paper-61.pdf)):**

**Author response:** We appreciate the insightful feedback from the reviewer specifically focusing on different type of embedding techniques and their role in our proposed methodology. The point-by-point response to the comments is given below:

**The use effect of word embeddings to encode fake news**

The use of word embeddings like Word2Vec and GloVe for fake news detection has been extensively studied due to their ability to capture semantic relationships between words. However, there are specific reasons why these embeddings were not included in the proposed research:

Word embeddings generate static word vectors, which fail to account for contextual nuances, such as changes in meaning based on surrounding text [1, 2]. While effective in supervised learning tasks, these are less suited for unsupervised methods like clustering, where the focus is on structural and statistical characteristics of the data. Approaches like TF-IDF provide term-specific weights that are more interpretable and better aligned with the objectives of the proposed research based on clustering [3]. Furthermore, word embeddings typically require additional preprocessing and fine-tuning to adapt them for domain-specific fake news detection. This process adds computational complexity and might not yield significant improvements for unsupervised learning methods [4]. The proposed research incorporated TF-IDF, being simpler and domain-agnostic, offers a scalable solution without the overhead of embedding model training or fine-tuning [5].

**Use of transformer embeddings to encode fake news**

Transformer embeddings are typically leveraged in supervised learning settings or fine-tuned for specific tasks. The proposed research incorporated unsupervised clustering techniques, where traditional representations like TF-IDF are often more effective and interpretable for capturing textual patterns [6]. Another key challenge associated with transformer-based models is their lack of transparency and interpretability. These models generate high-dimensional embeddings that can be difficult to analyze and interpret in the context of clustering, where understanding the relationship between data points is critical [7].

**How document embeddings are used to encode fake news**

Document embeddings are a powerful tool for encoding fake news by transforming textual data into numerical representations that capture the semantic and contextual essence of entire documents. Unlike traditional word-level embeddings, document embeddings aggregate information across sentences or paragraphs, making them particularly effective for tasks requiring an understanding of broader context.

However, the primary goal of this research is to analyze surface-level linguistic patterns for fake news detection. Therefore, traditional methods like TF-IDF were sufficient to capture the necessary textual features without the additional complexity of embedding-based approaches.

**Real-time architectures for fake news and harmful content detection and mitigation**

The proposed research primarily investigates unsupervised learning techniques, specifically GMM-GCO, a hybrid model to effectively identify fake news. Real-time architectures often involve supervised or semi-supervised methods, leveraging labeled data streams for real-time predictions. Integrating these techniques could deviate from the core aim of developing robust unsupervised methods for textual clustering. Furthermore, real-time feedback systems, such as those used in platforms like **ContCommRTD** or **StopHC** could demand infrastructure beyond the scope of the proposed research to implement and test these architectures

**Using social network features for fake news detection**

Real-time systems incorporate interdisciplinary components, such as user behavior analysis, network traffic monitoring, and large-scale content moderation, which is beyond the scope of our research. As the primary goal of this research is to analyze surface-level linguistic patterns for fake news detection.

**The effect of multilingual transformers on fake news detection**

Our study primarily focused on English-language datasets and align with several existing benchmark corpora in fake news detection studies. Since multilingual transformers like mBERT or XLM-R are designed to handle diverse languages, their inclusion is not necessary given the considered dataset's linguistic homogeneity. Multilingual models like mBERT and XLM-R have shown success in cross-lingual natural language understanding tasks, but their effectiveness in unsupervised or clustering techniques remains less explored [8, 9].

**Author action:**

We trust that these justifications align with the reviewer’s expectations and justify the exclusion of word or transformer embeddings in favor of simpler, domain-independent text representation techniques better aligned with the objectives of our proposed research.

**References:**

1. Mikolov T, Sutskever I, Chen K, Corrado GS, Dean J. Distributed representations of words and phrases and their compositionality. Advances in neural information processing systems. 2013;26.
2. Sun C, Yang Z, Luo L, Wang L, Zhang Y, Lin H, Wang J. A deep learning approach with deep contextualized word representations for chemical–protein interaction extraction from biomedical literature. IEEE Access. 2019 Oct 21;7:151034-46.
3. Sparck Jones K. A statistical interpretation of term specificity and its application in retrieval. Journal of documentation. 1972 Jan 1;28(1):11-21.
4. Farhangian F, Cruz RM, Cavalcanti GD. Fake news detection: Taxonomy and comparative study. Information Fusion. 2024 Mar 1;103:102140.
5. Pennington J, Socher R, Manning CD. Glove: Global vectors for word representation. InProceedings of the 2014 conference on empirical methods in natural language processing (EMNLP) 2014 Oct (pp. 1532-1543).
6. Park J, Park C, Kim J, Cho M, Park S. ADC: Advanced document clustering using contextualized representations. Expert Systems with Applications. 2019 Dec 15;137:157-66.
7. Li Y, Wang J, Dai X, Wang L, Yeh CC, Zheng Y, Zhang W, Ma KL. How does attention work in vision transformers? A visual analytics attempt. IEEE transactions on visualization and computer graphics. 2023 Mar 27;29(6):2888-900.
8. Conneau A, Baevski A, Collobert R, Mohamed A, Auli M. Unsupervised cross-lingual representation learning for speech recognition. arXiv preprint arXiv:2006.13979. 2020 Jun 24.
9. Pires T. How multilingual is multilingual BERT. arXiv preprint arXiv:1906.01502. 2019.

**Reviewer#1, Concern # 2. (What happens after detection? There are many solutions used for this proposed in the literature that also propose models for identifying fake news and mitigation.**

**Some related work that should be discussed (please respond individually):**

**- use of community detection for fake news network immunization [8] (e.g., https://doi.org/10.1016/j.jestch.2024.101728)**

**- use of weighted directed spanning trees for fake news detection and mitigation in real time [9] (e.g., https://doi.org/10.1109/ACCESS.2023.3331220)**

**- use of budget-based immunization algorithms to stop fake news from spreading [10] (e.g., https://doi.org/10.1145/3459637.3482481).):**

**Author response:** We sincerely appreciate the valuable feedback provided by the reviewer, a point by point response for each comment is given below:

**Use of community detection for fake news network immunization**

The proposed research primarily focuses on unsupervised clustering techniques and hybrid GMM methods for detecting fake news based on linguistic patterns in textual data. Community detection in fake news network immunization is more relevant to network-based methods, where relationships between social media accounts or information dissemination pathways are analyzed. This network-based focus falls outside the linguistic and clustering scope of your study. Community detection approaches rely on graph-based representations of social networks, where nodes represent users or sources and edges represent interactions or information flows. In contrast, our study employs textual data and uses TF-IDF vectorization combined with Gaussian Mixture Models (GMM) and Group Counseling Optimization (GCO). The lack of graph-structured data in the proposed research makes the integration of community detection techniques unsuitable.

Community detection frameworks often propose mitigation strategies like node immunization to curb the spread of fake news by identifying and neutralizing influential nodes. While valuable, such approaches require a detailed understanding of network dynamics, user influence, and propagation pathways, which is out of the scope of our research.

**Use of weighted directed spanning trees for fake news detection and mitigation in real time**

Our research primarily emphasizes the detection of fake news using unsupervised clustering techniques such as GMM, K-means, K-medoid and GCO-GMM, focusing on linguistic patterns in textual data. The proposed method focused on transforming text into numerical features (e.g., TF-IDF) and applying clustering algorithms to identify patterns. In contrast, approaches using weighted directed spanning tree (WDST) often operate on graph-based representations of information spread, focusing on the propagation of fake news across networks, which is beyond the scope of the proposed research.

Furthermore, our dataset does not include interaction or network related information (such as user engagement data, timestamps, or propagation paths) required for weighted directed spanning trees methods. The absence of such data makes it impractical to integrate WDST based methodologies into in the proposed research.

**Use of budget-based immunization algorithms to stop fake news from spreading**

Our proposed research is fundamentally designed to address fake news detection through **unsupervised clustering techniques**, particularly focusing on linguistic and textual patterns in news data. Budget-based immunization algorithms, which are primarily used in **network-based propagation control**, fall outside the scope of the proposed research. These algorithms focus on identifying key nodes (users or sources) in a social network to block or mitigate the spread of misinformation, requiring a network representation and interaction data.

Furthermore, our dataset does not include interaction or network related information (such as user engagement data, timestamps, or propagation paths) required for weighted directed spanning trees methods. The absence of such data makes it impractical to integrate WDST based methodologies in the proposed research.

**Author action:** We sincerely hope that these clarifications will meet the reviewers' expectations and effectively address their concerns.

**Reviewer#2, Concern # 3. (There are many methods for document similarity detection, not just clustering. The manuscript must be improved and it must discuss the following (please respond individually):**

**a. Document similarity using clustering for dimensionality reduction [11] (e.g., https://doi.org/10.1109/AQTR49680.2020.9129967)**

**b. Novel novel density-based clustering algorithms that can be used for topic modeling that should at least be mentioned, e.g., DenLAC [12], some even based on DBScan algorithms [13], for example, QuickDBScan and KDTreeDBSCAN [14].**

**c. Community Detection-based methods for document similarity [15].**

**d. Similarity based on context-aware document clustering [16].**

**e. How are the metrics for encoding the textual data selected? Please compare with other methods, you can start from [17] (e.g.,** [**http://doi.org/10.1109/SYNASC.2016.055)**](http://doi.org/10.1109/SYNASC.2016.055))**).**

**a. Document similarity using clustering for dimensionality reduction**

The primary objective of the proposed research is to optimize GMM parameters using GCO for improved clustering results and efficiently detection of fake news. Clustering for dimensionality reduction, while valuable, does not directly contribute to the parameter optimization goals.

Furthermore the proposed research already employs TF-IDF and PCA for feature extraction and dimensionality reduction (Please see the highlighted content under 2. 1. 8 heading on page 10 and 11). These techniques have strong theoretical and empirical implications in fake news detection, and introducing clustering for dimensionality reduction might not add substantial value over the current methods.

The focus on fake news detection through clustering assumes a direct clustering algorithm application. Clustering for dimensionality reduction serves a more generalized purpose and could dilute the research scope.

**b. Novel density-based clustering algorithms that can be used for topic modeling that should at least be mentioned, e.g., DenLAC [12], some even based on DBScan algorithms [13], for example, QuickDBScan and KDTreeDBSCAN [14].**

Our prior experience with DBSCAN yielded unsatisfactory results in terms of clustering accuracy for fake news detection. Although these newer density-based algorithms (QuickDBScan, KDTreeDBSCAN and DenLAC) may offer improvements, the reason to exclude them was likely based on the failure of DBSCAN to meet the requirements for our specific dataset and the added complexity these algorithms could introduce without guaranteed improvements.

The primary focus of the proposed research is on optimizing the parameters of the Gaussian Mixture Model (GMM) using the Group Counseling Optimization (GCO) method. As GMM offers a more probabilistic approach to clustering, that has the potential to capture the uncertainty of fake news data more effectively, it was deemed more suitable for our goals than attempting to integrate another clustering method that may require additional optimization efforts.

**c. Community Detection-based methods for document similarity**

The proposed research prioritizes optimizing GMM for clustering due to its probabilistic nature. While community detection offers graph-based insights, it differs significantly in its underlying assumptions and might not align directly with our optimization framework.

Furthermore, our dataset does not include interaction or network related information (such as user engagement data, timestamps, or propagation paths) required for graph-based methods. The absence of such data makes it impractical to integrate graph based methodologies in the proposed research.

**d. Similarity based on context-aware document clustering**

The use of word embeddings like Word2Vec and GloVe for fake news detection has been extensively studied due to their ability to capture semantic relationships between words. However, there are specific reasons why these embeddings were not included in the proposed research:

Word embeddings generate static word vectors, which fail to account for contextual nuances, such as changes in meaning based on surrounding text [1, 2]. While effective in supervised learning tasks, word embeddings are less suited for unsupervised methods like clustering, where the focus is on structural and statistical characteristics of the data. Approaches like TF-IDF provide term-specific weights that are more interpretable and better aligned with the objectives of unsupervised clustering [3]. Word embeddings typically require additional preprocessing and fine-tuning to adapt them for domain-specific fake news detection. This process adds computational complexity and might not yield significant improvements for unsupervised learning methods [4]. TF-IDF, being simpler and domain-agnostic, offers a scalable solution without the overhead of embedding model training or fine-tuning [5].

**e. How are the metrics for encoding the textual data selected? Please compare with other methods**

The choice of metrics for encoding textual data depends on several factors such as the nature of the dataset, the computational requirements, the application domain (e.g., clustering, classification), and the specific challenges the data presents. Different methods for encoding textual data vary in complexity and utility based on their intended applications Bag-of-Words (BoW), TF-IDF (Term Frequency-Inverse Document Frequency, Word embeddings, contextual embeddings and Latent Dirichlet Allocation (LDA). The Bag-of-Words (BoW) approach encodes word frequency without considering the order, offering simplicity and interpretability. TF-IDF improves upon BoW by weighing words based on their importance across documents, making it effective for relevance balancing but though these both suffer from ignoring context and producing sparse representations. Word embeddings, such as Word2Vec and GloVe, generate dense vectors capturing semantic relationships between words, effectively representing similarity but failing to encode the context of entire documents. In contrast, contextual embeddings like BERT and RoBERTa account for word context within sentences, offering deep semantic understanding but at the cost of high computational resources and reliance on pre-trained models. Finally, Latent Dirichlet Allocation (LDA), a topic modeling method, represents documents as distributions over topics, making it excellent for uncovering latent structures; however, it lacks detailed semantic encoding and demands extensive preprocessing. Each method has distinct strengths and weaknesses, making the selection task-specific and resource-dependent.

In the proposed, Bag-of-Words (BoW) and TF-IDF were likely chosen due to their simplicity, effectiveness, and alignment with your objectives. This simplicity makes it suitable for unsupervised techniques where interpretability is crucial, as it allows clustering algorithms to focus on frequently occurring terms without being biased by complex structures. TF-IDF builds upon BoW enhances the quality of clustering results by emphasizing informative features. Furthermore, it also ensures that the representation remains computationally manageable while retaining key patterns.

**References:**

1. Mikolov T, Sutskever I, Chen K, Corrado GS, Dean J. Distributed representations of words and phrases and their compositionality. Advances in neural information processing systems. 2013;26.
2. Sun C, Yang Z, Luo L, Wang L, Zhang Y, Lin H, Wang J. A deep learning approach with deep contextualized word representations for chemical–protein interaction extraction from biomedical literature. IEEE Access. 2019 Oct 21;7:151034-46.
3. Sparck Jones K. A statistical interpretation of term specificity and its application in retrieval. Journal of documentation. 1972 Jan 1;28(1):11-21.
4. Farhangian F, Cruz RM, Cavalcanti GD. Fake news detection: Taxonomy and comparative study. Information Fusion. 2024 Mar 1;103:102140.
5. Pennington J, Socher R, Manning CD. Glove: Global vectors for word representation. InProceedings of the 2014 conference on empirical methods in natural language processing (EMNLP) 2014 Oct (pp. 1532-1543).

**Reviewer 1, Concern # 4. (An in-depth exploratory data analysis is required.)**

**Author response:** We appreciate the reviewer’s suggestion. In the main manuscript file, an in-depth exploratory data analysis has already been conducted and documented under the Methodology section. Specifically, the EDA includes:

The preprocessing phase ensures clean and consistent data for analysis. Text was converted to lowercase to maintain uniformity and avoid inconsistencies arising from case differences. Tokenization split text into smaller units (words or phrases) for easier manipulation. Non-alphanumeric characters, stop words, and punctuation were removed to reduce noise and focus on semantically meaningful terms. Stemming (using the Porter Stemmer) normalized words to their root forms, consolidating similar terms and reducing vocabulary size for more effective clustering and similarity analysis.

Optimal cluster numbers were determined using techniques like the Elbow Method and BIC Statistics. These methods assess clustering quality by evaluating within-cluster variance, inter-cluster separation, and overall structure, aiding in model selection. Subsequently, term-frequency matrices were generated for textual data, alongside dimensionality reduction techniques like TF-IDF to simplify feature spaces. Visualizations such as word clouds were created to highlight significant terms and patterns, offering insights into the data's thematic structure. The proposed method also applied PCA to reduce the feature space, improving computational efficiency and clustering accuracy (Please see the highlighted content under 2. 1. 8 heading on page 10 and 11).. This method also facilitated better visualization of clustering results, enhancing the interpretability of the data and models.

**Author Action:** We believe that this level of EDA sufficiently supports the objectives of our study, providing both foundational understanding and practical insights for the proposed approach. If the reviewer feels additional EDA components are necessary, we would welcome specific suggestions to incorporate further improvements.

**Reviewer 1, Concern # 5. (The experiments should be improved (please respond individually to each point in the answer to reviewers)**

a. **There is no hyperparameter tuning for the proposed model.**

We thank the reviewer for highlighting this aspect. We would like to clarify that hyper-parameter tuning was conducted as part of our methodology, and details have been included in the revised manuscript. Specifically:

For K-means and K-medoids, the optimal number of clusters (𝑘) was determined using robust methods such as the Elbow Method. This approach ensures the balance between intra-cluster compactness and inter-cluster separation. Bayesian Information Criterion (BIC) was utilized to select the optimal number of components and covariance type for Gaussian Mixture Model (GMM) (Please see page no. 17), ensuring a trade-off between model fit and complexity. We also incorporated Group Counseling Optimization (GCO) algorithm to automate the hyper parameter tuning process for GMM. Furthermore, each tuning process was validated through three evaluation measures including silhouette score, adjusted rand index and purity score. These evaluation metrics help in assessing the effectiveness of the unsupervised techniques and provide quantitative measures of its performance.

**b. There is no time performance evaluation.**

**Author response:** In response to the comment regarding time performance evaluation, we have conducted an analysis of the time complexity for the proposed hybrid model as highlighted in the main manuscript file (Please see the highlighted content on page no. 20).

**Author action:** The manuscript has also been revised accordingly as reflected in the main manuscript file highlighted under the experimental setup section in the last paragraph of page 19 and 20 of the main manuscript file.

**c. The experiments are done on relatively small datasets. How does the proposed model perform on a larger dataset?**

**Author response:** The performance of the proposed model on larger datasets would depend on the scalability of both the GMM and the GCO. GMM's time complexity increases with the number of data points and components, but techniques like mini-batch EM or variational inference can help manage larger datasets (Bishop, 2006). While GCO optimizes high-dimensional data and is efficient, larger datasets may increase computational demands. Thus, the model may require additional optimization to scale effectively for larger datasets.

**Reference:**

1. Bishop CM, Nasrabadi NM. Pattern recognition and machine learning. New York: springer; 2006 Aug 17.

**d. There is no comparison with state-of-the-art models.**

**Author response:** We sincerely appreciate the valuable feedback provided by the reviewer on this aspect.

**Author action:** While our research focuses on a novel hybrid approach combining Group Counseling Optimization (GCO) with Gaussian Mixture Models (GMM) for fake news detection, we understand the importance of situating our model within the context of existing state-of-the-art techniques. Therefore, the proposed hybrid model was rigorously compared against traditional clustering algorithms, including Gaussian Mixture Models (GMM), K-means, and K-medoids, using the same news dataset. This comparative analysis aimed to evaluate the effectiveness and robustness of our approach in detecting fake news. (Please see the detail on page 27 and 28, as highlighted in the main manuscript file).

**Reviewer 1, Concern # 6. (The conclusions are very shallow.)**

**Author response:** We acknowledge the reviewer's feedback regarding the conclusion's depth.

**Author action:** To address this, we have revised and summarize the key findings, highlighting the proposed GCO-GMM hybrid model's superior performance in fake news detection compared to traditional clustering methods like GMM, K-means, and K-medoids. We also described the broader implications of these results for real-world applications, particularly in social media monitoring and content moderation. Additionally, we have also suggested future research directions, including exploring larger datasets, further optimization of the model, and integration with advanced techniques such as transformer-based models.

**Reviewer 1, Concern # 7. (What are the limitations of this study?)**

**Author response:** We sincerely appreciate the valuable feedback provided by the reviewer, particularly the limitation of the proposed research.

**Author Action:** In response to reviewer’s valuable suggestion regarding the limitation of the proposed research, we have discussed some limitation in the main manuscript file (Please see the highlighted detail on the last paragraph of page no 28).

**Reviewer 1, Concern # 8. (For reproducibility purposes, the authors should make the code publicly available. Without publicly available source code, models, and datasets, a shadow of doubt can fall on the results of this work. How can anyone check if these results are real or made up without the code, models, and datasets? )**

**Author response:** We appreciate the reviewer's concern regarding the reproducibility of our results. To ensure transparency and facilitate independent validation, we have made the source code, trained models, and dataset used in our study publicly available on GitHub. The GitHub repository contains all necessary files.

**Author Action:** The repository link has been included in the revised manuscript under the "Data and Code Availability" section.

**Reviewer 1, Concern # 9. (Please proofread and spell-check the article before resubmitting.**

**Author response:** We appreciate the reviewer's observation regarding the need for proofreading and spell-checking.

**Author Action:** To address this, we have thoroughly reviewed the manuscript for grammatical errors, spelling issues, and etc.

**Reviewer#2, Concern # 1. (Why choose TF-IDF for encoding the documents?**

**Why not word embeddings [1], transformers [2], or document embeddings [3]? )**

**Author response:** We appreciate the insightful feedback from the reviewer specifically focusing on different type of embedding techniques and their role in our proposed methodology.

**The use effect of word embeddings to encode fake news**

The use of word embeddings like Word2Vec and GloVe for fake news detection has been extensively studied due to their ability to capture semantic relationships between words. However, there are specific reasons why these embeddings were not included in the proposed research:

Word embeddings generate static word vectors, which fail to account for contextual nuances, such as changes in meaning based on surrounding text [1, 2]. While effective in supervised learning tasks, these are less suited for unsupervised methods like clustering, where the focus is on structural and statistical characteristics of the data. Approaches like TF-IDF provide term-specific weights that are more interpretable and better aligned with the objectives of the proposed research based on clustering [3]. Furthermore, word embeddings typically require additional preprocessing and fine-tuning to adapt them for domain-specific fake news detection. This process adds computational complexity and might not yield significant improvements for unsupervised learning methods [4]. The proposed research incorporated TF-IDF, being simpler and domain-agnostic, offers a scalable solution without the overhead of embedding model training or fine-tuning [5].

**Use of transformer embeddings to encode fake news**

Transformer embeddings are typically leveraged in supervised learning settings or fine-tuned for specific tasks. The proposed research incorporated unsupervised clustering techniques, where traditional representations like TF-IDF are often more effective and interpretable for capturing textual patterns [6]. Another key challenge associated with transformer-based models is their lack of transparency and interpretability. These models generate high-dimensional embeddings that can be difficult to analyze and interpret in the context of clustering, where understanding the relationship between data points is critical [7].

**How document embeddings are used to encode fake news**

**Author response:** Document embeddings are a powerful tool for encoding fake news by transforming textual data into numerical representations that captures the semantic and contextual essence of entire documents. Unlike traditional word-level embeddings, document embeddings aggregate information across sentences or paragraphs, making them particularly effective for tasks requiring an understanding of broader context.

However, the primary goal of this research is to analyze surface-level linguistic patterns for fake news detection. Therefore, traditional methods like TF-IDF were sufficient to capture the necessary textual features without the additional complexity of embedding-based approaches.

**Author action:** We sincerely hope that these clarifications will meet the reviewers' expectations and effectively address their concerns.

**Reviewer#2, Concern #2. (Does the method work in real-time? Please compare with existing methods and architectures that work in real-time [4] or [5].)**

**Author response:** The proposed research primarily investigates unsupervised learning techniques, specifically GMM-GCO, a hybrid model to effectively identify fake news. Real-time architectures often involve supervised or semi-supervised methods, leveraging labeled data streams for real-time predictions. Integrating these techniques could deviate from the core aim of developing robust unsupervised methods for textual clustering. Furthermore, real-time feedback systems, such as those used in platforms like **ContCommRTD** or **StopHC** could demand infrastructure beyond the scope of the proposed research to implement and test these architectures

**Reviewer#2, Concern #3. (What happens when encoding more than the textual content? For example, if using social network features for fake news detection [6])**

**Author response:** Real-time systems incorporate interdisciplinary components, such as user behavior analysis, network traffic monitoring, and large-scale content moderation, which is beyond the scope of our research. As the primary goal of this research is to analyze surface-level linguistic patterns for fake news detection.

**Reviewer#2, Concern # 4. (How do you deal with multilingual textual data? Compared with existing methods [7].)**

**Author response:** Our study primarily focused on English-language datasets and align with several existing benchmark corpora in fake news detection studies. Since multilingual transformers like mBERT or XLM-R are designed to handle diverse languages, their inclusion is not necessary given the considered dataset's linguistic homogeneity. Multilingual models like mBERT and XLM-R have shown success in cross-lingual natural language understanding tasks, but their effectiveness in unsupervised or clustering techniques remains less explored [1, 2, 3].

**Author action:**

We trust that these additions align with your expectations and justify the exclusion of word or transformer embeddings in favor of simpler, domain-independent text representation techniques better aligned with the objectives of our proposed research.

References:

1. Conneau A, Baevski A, Collobert R, Mohamed A, Auli M. Unsupervised cross-lingual representation learning for speech recognition. arXiv preprint arXiv:2006.13979. 2020 Jun 24.
2. Pires T. How multilingual is multilingual BERT. arXiv preprint arXiv:1906.01502. 2019.
3. Park J, Park C, Kim J, Cho M, Park S. ADC: Advanced document clustering using contextualized representations. Expert Systems with Applications. 2019 Dec 15;137: 157-66.

**Reviewer#2, Concern # 5. (What's next after detection? In the current literature, many works propose models for fake news detection and mitigation, e.g., community-based [8], trees-based [9], or pre-emptive [10] algorithms. The authors must discuss some direction at least in future work.)**

**Author response:** Our research primarily emphasizes the detection of fake news using unsupervised clustering techniques such as GMM, K-means, K-medoid and GCO-GMM, focusing on linguistic patterns in textual data. The proposed method focused on transforming text into numerical features (e.g., TF-IDF) and applying clustering algorithms to identify patterns. In contrast, approaches using weighted directed spanning tree (WDST) often operate on graph-based representations of information spread, focusing on the propagation of fake news across networks, which is beyond the scope of the proposed research.

Furthermore, our dataset does not include interaction or network related information (such as user engagement data, timestamps, or propagation paths) required for weighted directed spanning trees methods. The absence of such data makes it impractical to integrate WDST based methodologies into in the proposed research.

**Author Action:** In response to reviewer’s valuable suggestion regarding the future work of the proposed research, we have discussed some limitation as the future directions in the main manuscript file (Please see the highlighted detail on the last paragraph of page no 28).

**Reviewer#2, Concern # 6. (Why use only classic clustering methods for your approach? There are many clustering methods that employ dimensionality reduction [11]. Please improve the manuscript and compare your methods with these methods.)**

**Author response:** The primary objective of the proposed research is to optimize GMM parameters using GCO for improved clustering results and efficiently detection of fake news. Clustering for dimensionality reduction, while valuable, does not directly contribute to the parameter optimization goals.

Furthermore the proposed research already employs TF-IDF and PCA for feature extraction and dimensionality reduction (Please see the highlighted content under 2. 1. 8 heading on page 10 and 11). These techniques have strong theoretical and empirical implications in fake news detection, and introducing clustering for dimensionality reduction might not add substantial value over the current methods.

The focus on fake news detection through clustering assumes a direct clustering algorithm application. Clustering for dimensionality reduction serves a more generalized purpose and could dilute the research scope.

**Reviewer#2, Concern # 7. (On the topic of clustering, why not use density-based algorithms [12] (e.g., DenLAC) or DBScan algorithms [13], for example, QuickDBScan and KDTreeDBSCAN [14]. Please improve the manuscript and compare your methods with these methods.)**

**Author response:** Our prior experience with DBSCAN yielded unsatisfactory results in terms of clustering accuracy for fake news detection. Although these newer density-based algorithms (QuickDBScan, KDTreeDBSCAN and DenLAC) may offer improvements, the reason to exclude them was likely based on the failure of DBSCAN to meet the requirements for our specific dataset and the added complexity these algorithms could introduce without guaranteed improvements.

The primary focus of the proposed research is on optimizing the parameters of the Gaussian Mixture Model (GMM) using the Group Counseling Optimization (GCO) method. As GMM offers a more probabilistic approach to clustering, that has the potential to capture the uncertainty of fake news data more effectively, it was deemed more suitable for our goals than attempting to integrate another clustering method that may require additional optimization efforts.

**Reviewer#2, Concern # 8. (Community Detection-based methods for document similarity [15] are also used for clustering documents. Please improve the manuscript and compare your methods with these methods.)**

**Author response:** The proposed research prioritizes optimizing GMM for clustering due to its probabilistic nature. While community detection offers graph-based insights, it differs significantly in its underlying assumptions and might not align directly with our optimization framework.

Furthermore, our dataset does not include interaction or network related information (such as user engagement data, timestamps, or propagation paths) required for graph-based methods. The absence of such data makes it impractical to integrate graph based methodologies in the proposed research.

**Reviewer#2, Concern # 9. (Why not use topic modeling for clustering documents? There are many context-aware document topic modeling algorithms [16] used in the literature for document clustering. Please improve the manuscript and compare your methods with these methods.)**

**Author response:** The use of word embeddings like Word2Vec and GloVe for fake news detection has been extensively studied due to their ability to capture semantic relationships between words. However, there are specific reasons why these embeddings were not included in the proposed research:

Word embeddings generate static word vectors, which fail to account for contextual nuances, such as changes in meaning based on surrounding text [1, 2]. While effective in supervised learning tasks, word embeddings are less suited for unsupervised methods like clustering, where the focus is on structural and statistical characteristics of the data. Approaches like TF-IDF provide term-specific weights that are more interpretable and better aligned with the objectives of unsupervised clustering [3]. Word embeddings typically require additional preprocessing and fine-tuning to adapt them for domain-specific fake news detection. This process adds computational complexity and might not yield significant improvements for unsupervised learning methods [4]. TF-IDF, being simpler and domain-agnostic, offers a scalable solution without the overhead of embedding model training or fine-tuning [5].

**References:**

1. Mikolov T, Sutskever I, Chen K, Corrado GS, Dean J. Distributed representations of words and phrases and their compositionality. Advances in neural information processing systems. 2013;26.
2. Sun C, Yang Z, Luo L, Wang L, Zhang Y, Lin H, Wang J. A deep learning approach with deep contextualized word representations for chemical–protein interaction extraction from biomedical literature. IEEE Access. 2019 Oct 21;7:151034-46.
3. Sparck Jones K. A statistical interpretation of term specificity and its application in retrieval. Journal of documentation. 1972 Jan 1;28(1):11-21.
4. Farhangian F, Cruz RM, Cavalcanti GD. Fake news detection: Taxonomy and comparative study. Information Fusion. 2024 Mar 1;103:102140.
5. Pennington J, Socher R, Manning CD. Glove: Global vectors for word representation. InProceedings of the 2014 conference on empirical methods in natural language processing (EMNLP) 2014 Oct (pp. 1532-1543).

**Reviewer#2, Concern # 10. (On what basis was TF-IDF selected for encoding the textual data? There are many works that discuss different weighting schemes for document clustering [17]. Please improve the manuscript and compare your methods with these methods.)**

Author response: We appreciate the reviewer's observation regarding the selection of TF-IDF for encoding textual data and the suggestion to compare it with alternative weighting schemes. The choice of metrics for encoding textual data is pivotal in determining the performance and suitability of the model.

Author action: In the revised manuscript, we have expanded the discussion to include a comparison among TF-IDF and different encoding schemes (Please see the highlighted content under 2. 1. 7 heading on page 9).

**Reviewer#2, Concern # 11. (The datasets are not properly presented. Please improve the manuscript with an exploratory data analysis.)**

**Author response:** We appreciate the reviewer’s suggestion. In the main manuscript file, an in-depth exploratory data analysis has already been conducted and documented under the Methodology section. Specifically, the EDA includes:

The preprocessing phase ensures clean and consistent data for analysis. Text was converted to lowercase to maintain uniformity and avoid inconsistencies arising from case differences. Tokenization split text into smaller units (words or phrases) for easier manipulation. Non-alphanumeric characters, stop words, and punctuation were removed to reduce noise and focus on semantically meaningful terms. Stemming (using the Porter Stemmer) normalized words to their root forms, consolidating similar terms and reducing vocabulary size for more effective clustering and similarity analysis.

Optimal cluster numbers were determined using techniques like the Elbow Method and BIC Statistics. These methods assess clustering quality by evaluating within-cluster variance, inter-cluster separation, and overall structure, aiding in model selection. Subsequently, term-frequency matrices were generated for textual data, alongside dimensionality reduction techniques like TF-IDF to simplify feature spaces. Visualizations such as word clouds were created to highlight significant terms and patterns, offering insights into the data's thematic structure. The proposed method also applied PCA to reduce the feature space, improving computational efficiency and clustering accuracy (Please see the highlighted content under 2. 1. 8 heading on page 10 and 11).. This method also facilitated better visualization of clustering results, enhancing the interpretability of the data and models.

**Author action:** We believe that this level of EDA sufficiently supports the objectives of our study, providing both foundational understanding and practical insights for the proposed approach. If the reviewer feels additional EDA components are necessary, we would welcome specific suggestions to incorporate further improvements.

**Reviewer#2, Concern # 12. (The experiments are missing a hyperparameter tuning analysis.)**

**Author response:** We thank the reviewer for highlighting this aspect. We would like to clarify that hyperparameter tuning was conducted as part of our methodology, and details have been included in the revised manuscript.

**Author action:** Specifically, for K-means and K-medoids, the optimal number of clusters (𝑘) was determined using robust methods such as the Elbow Method. This approach ensures the balance between intra-cluster compactness and inter-cluster separation. Bayesian Information Criterion (BIC) was utilized to select the optimal number of components and covariance type for Gaussian Mixture Model (GMM) (Please see last paragraph of page 16 and 17), ensuring a trade-off between model fit and complexity. We also incorporated Group Counseling Optimization (GCO) algorithm to automate the hyper parameter tuning process for GMM. Furthermore, each tuning process was validated through three evaluation measures including silhouette score, adjusted rand index and purity score. These evaluation metrics help in assessing the effectiveness of the unsupervised techniques and provide quantitative measures of its performance.

**Reviewer#2, Concern # 13. (The experiments are missing a time performance evaluation.)**

**Author response:** In response to the comment regarding time performance evaluation, we have conducted an analysis of the time complexity for the proposed hybrid model as highlighted in the main manuscript file (Please see the highlighted content on page 20).

**Author action:** The manuscript has also been revised accordingly as reflected in the main manuscript file highlighted under the experimental setup section in the last paragraph of page no. 20 of the main manuscript file.

**Reviewer#2, Concern # 14. (The size of the datasets is limited, how does the proposed model perform on a large dataset?)**

**Author response:** The performance of the proposed model on larger datasets would depend on the scalability of both the GMM and the GCO. GMM's time complexity increases with the number of data points and components, but techniques like mini-batch EM or variational inference can help manage larger datasets (Bishop, 2006). While GCO optimizes high-dimensional data and is efficient, larger datasets may increase computational demands. Thus, the model may require additional optimization to scale effectively for larger datasets.

**Reference:**

1. Bishop CM, Nasrabadi NM. Pattern recognition and machine learning. New York: springer; 2006 Aug 17.

**Reviewer#2, Concern # 15. (Improve the manuscript by comparing your method with existing state-of-the-art models.)**

**Author response:** We sincerely appreciate the valuable feedback provided by the reviewer on this aspect.

**Author action:** While our research focuses on a novel hybrid approach combining Group Counseling Optimization (GCO) with Gaussian Mixture Models (GMM) for fake news detection, we understand the importance of situating our model within the context of existing state-of-the-art techniques. Therefore, the proposed hybrid model was rigorously compared against traditional clustering algorithms, including Gaussian Mixture Models (GMM), K-means, and K-medoids, using the same news dataset. This comparative analysis aimed to evaluate the effectiveness and robustness of our approach in detecting fake news. (Please see the detail on page 28, as highlighted in the main manuscript file).

**Reviewer#2, Concern # 16. (The conclusions must be improved.)**

**Author response:** We acknowledge the reviewer's feedback regarding the conclusion's depth.

**Author action:** To address this, we have revised and summarize the key findings, highlighting the proposed GCO-GMM hybrid model's superior performance in fake news detection compared to traditional clustering methods like GMM, K-means, and K-medoids. We also described the broader implications of these results for real-world applications, particularly in social media monitoring and content moderation. Additionally, we have also suggested future research directions, including exploring larger datasets, further optimization of the model, and integration with advanced techniques such as transformer-based models.

**Reviewer#2, Concern # 17. (A limitation section is missing. What are the limitations of your methods?)**

**Author response:** We sincerely appreciate the valuable feedback provided by the reviewer, particularly the limitation of the proposed research.

**Author Action:** In response to reviewer’s valuable suggestion regarding the limitation of the proposed research, we have discussed some limitation in the main manuscript file (Please see the highlighted detail on the last paragraph of page no 28).

**Reviewer#2, Concern # 18. (The authors should make the code publicly available for reproducibility purposes.)**

**Author response:** We appreciate the reviewer's concern regarding the reproducibility of our results. To ensure transparency and facilitate independent validation, we have made the source code, trained models, and dataset used in our study publicly available on GitHub. The GitHub repository contains all necessary files.

**Author Action:** The repository link has been included in the revised manuscript under the "Data and Code Availability" section.

**Reviewer#2, Concern # 19. (Before resubmitting, I recommend proofreading and spell-checking the article.)**

**Author response:** We appreciate the reviewer's observation regarding the need for proofreading and spell-checking.

**Author Action:** To address this, we have thoroughly reviewed the manuscript for grammatical errors, spelling issues, and etc.
