# Supplementary material for: Unsupervised fake news detection on social media using hybrid Gaussian Mixture Model
Source: PLoS One. 2025 Aug 18;20(8):e0330421. doi: 10.1371/journal.pone.0330421 (PMC12360590; doi:10.1371/journal.pone.0330421)
Supplement: Attachment — Submitted filename: Response-to-Reviewers 23-4-25.docx [file pone.0330421.s003.docx]

**Original Manuscript ID:** PONE-D-24-50144

**Original Article Title: “**Unsupervised fake news detection on social media using hybrid gaussian mixture model”

**To:** PLOS ONE Editor

**Re:** Response to reviewers

Dear Editor,

Thank you for allowing a submission of our revised manuscript, with an opportunity to address the reviewers’ comments.

We are uploading (a) our point-by-point response to the comments (below) (response to reviewers, under “Response-to-Reviewers Files*”*), (b) an updated manuscript with yellow highlighting indicating changes (as “Revised Article with Changes Highlighted*”*), and (c) a clean updated manuscript without highlights (“Manuscript”*).*

Best regards,

**Reviewer#1, Concern # 1 (Abstract is filled by useless background text starting from the "advent of World Wide Web" and its revolutionary rise making me doubt which century this paper was written. There are also typos (reserch) indicating that nobody has put serious efforts for the writing. This is amazing since there are 8 authors in the paper. It takes just couple of sentences to define the research question (fake news detection), and the proposed approach (unsupervised clustering) and the novelty (measure to select the number of clustrers).**

**Author response:** Thank you for your valuable feedback. We acknowledge that the abstract could be more concise and focused. The background section was intended to provide context about the evolution of the internet and its implications on data-driven challenges, but we understand that it may have been overly verbose. We have revised the abstract to remove unnecessary background details, ensuring it directly highlights the research question, the proposed approach, and its novelty.

We also appreciate your observation about the typo and we have corrected all such errors to maintain the quality of the paper. Furthermore, we have ensured that the revised abstract is clear, focused, and effectively conveys the core contributions of our research.

**Reviewer#4, Concern # 2. (Introduction focus on superficial literature review, and near the end, suddenly jumping from the background to explaining that Gaussian mixture models has been widely used modeling dynamic systems without telling what the dynamic system is and how it relates to the previous text.):**

To address this concern, we have revised the Introduction section to provide a more focused and critical review of related work in fake news detection, particularly highlighting the limitations of existing supervised and unsupervised approaches.

Moreover, we have clarified the reference to dynamic systems by explicitly explaining how fake news detection involves the analysis of evolving data patterns such as changes in misinformation themes, propagation behaviors, and user engagement, which align conceptually with the characteristics of dynamic systems. This connection provides the rationale for employing GMMs, a well-suited model for such complex problem and continuously changing data distributions.

**Author action:** The manuscript has also been revised accordingly as reflected in the main manuscript file highlighted under the Introduction section in the last paragraph of page 4 and 5 of the main manuscript file.

**Reviewer#4, Concern # 3. (Section 2 is non-innovatively titles as "Proposed methodology" covering everything from pre-processing (TF-IDF is hardly pre-processing anymore), to Gaussian mixture models and selecting the number of clusters including four levels of sections showing the complete failure to organize the writing properly. The results then follow in section which is not even numbered.):**

**Author response:** Thank you for highlighting this important issue. We acknowledge that the title "Proposed Methodology" lacks specificity and does not adequately reflect the structure or content of the section. In response, we have reorganized Section 2 to improve clarity and logical flow. The section is now divided into clearly labeled subsections, each focusing on a distinct component of the study, including data representation, classical clustering techniques, and determining the optimal number of components for clustering algorithms. Additionally, we have refined the use of headings to avoid unnecessary nesting and ensure a consistent structure throughout.

Regarding TF-IDF, we agree that it is more appropriately categorized as a feature extraction or vectorization method rather than pre-processing. We have revised the terminology accordingly to reflect this.

Finally, the Results section has been properly numbered and positioned to follow seamlessly from the Methodology section, ensuring consistency and improving the overall readability of the paper.

**Author action:** We sincerely hope that these clarifications will meet the reviewers' expectations and effectively address their concerns.

**Reviewer#4, Concern # 4. (GCO is claimed to be used to solve the hyperparameters like the number of components and converging threshold but later Elbow method and BIC are introduced anyway. Why? Elbow method seems to refer calculating first derivative (delta), which is not sufficient to alone for the task ).**

Thank you for your insightful observation. We acknowledge that the initial description of the Group Counseling Optimizer (GCO) may have caused confusion regarding its role in the proposed methodology. Basically, GCO is employed as the primary mechanism for optimizing key hyper-parameters most notably, the number of components in the Gaussian Mixture Model (GMM) and the convergence threshold within the hybrid clustering framework.

The inclusion of the Elbow Method and Bayesian Information Criterion (BIC) serves a comparative purpose rather than a conflicting one. Specifically, these conventional techniques were used to establish baseline values for the number of clusters/components in the traditional implementations of K-means, K-medoids, and GMM, respectively. This allows us to evaluate the performance of the proposed GCO-based optimization against widely accepted selection strategies.

We agree with the reviewer’s point that the Elbow Method, especially when relying solely on first derivatives, may not always provide a reliable or conclusive estimate of the optimal number of clusters. This further reinforces the need for a more adaptive and intelligent approach, such as GCO, which can explore the hyper-parameters more effectively and avoid reliance on heuristic or visual methods.

**Author action:** In the revised manuscript, we have clarified this distinction and made explicit the rationale behind including these traditional methods for comparison purposes only, to demonstrate the added value and robustness of our proposed hybrid approach. The manuscript has also been revised accordingly and highlighted on page 18 of the main manuscript file.

**Reviewer 4, Concern # 5. (The more essential are missing from the paper. Why (and how) we can expect to detect fake news from the clustering result, or Gaussian mixture models? None of the pictures show the true/fake labels for the points, only there cluster id.)**

**Author response:**

Thank you for raising this important point. We acknowledge that the connection between clustering results and the identification of fake news was not sufficiently emphasized in the manuscript. In unsupervised settings, such as ours, clustering algorithms including GMM aim to group data points based on similarities in their feature representations. The underlying assumption is that fake and real news articles exhibit distinct linguistic or structural patterns that can be captured in the feature space. By identifying clusters that correspond to these hidden patterns, we aim to differentiate between authentic and fake news without relying on labeled data.

To strengthen this connection, we have revised the manuscript to explain how cluster assignments are post-evaluated against ground truth labels (available for results validation) using metrics such as silhouette score, adjusted Rand index (ARI), and purity. These metrics help assess how well the discovered clusters align with actual fake/real categories.

**Reviewer 4, Concern # 6. (Figures are missing from the paper but included separately making it hard to follow. The document is also mixed by review reports, responses, two versions of the paper, in seemingly random order. Not sure if this is automated or author organized order of the material, but it just does not work well.)**

Thank you for pointing this out. We sincerely apologize for the confusion caused by the disorganized submission format. The inclusion of review reports, responses, and multiple versions of the manuscript in a non-sequential order was unintentional and may have resulted from an automated merge of files during submission.

We also understand that separating figures from the main manuscript may have made it harder to follow the content. However, the figures were uploaded separately in accordance with the journal’s submission requirements. We ensured that each figure is clearly referenced and appropriately captioned within the manuscript to maintain coherence between the text and the visuals.

**Reviewer 5, Concern # 1. (Balancing the data: were class balancing algorithms used for this purpose or data were chosen based on a random number generator or similar? Please, clarify. If that is the case, why weren't class balancing algorithms used? Also, what parts of the data are used: are these vectors of words and/or TF-IDF values?)**

**Author response:** Thank you for your valuable comment. In the proposed research, class balancing was not performed using a particular algorithm such as SMOTE. Instead, to ensure equitable representation across categories and prevent class imbalance during evaluation, we randomly selected 839 instances from each category without replacement, creating a balanced dataset as already depicted in the main manuscript file on page no. 7 under the data representation heading. This approach helps to maintain a uniform class distribution while preserving the integrity of the data.

As the study focuses on an unsupervised approach, labels were not used during the clustering process itself. However, a balanced dataset was important for fair post-clustering evaluation using metrics such as ARI and purity, which compare the clustering outcomes to the actual labels.

Furthermore, the data instances used for clustering are based on TF-IDF vector representations of the news articles. Each document is converted into a high-dimensional numerical vector capturing the relative importance of words across the corpus, which serves as the input for all clustering algorithms including GMM, K-means, and K-medoids.

**Reviewer 5, Concern # 2. (PCA analysis: please clarify the input data for this. What is the dimension of the post-processed data? Are only the words in form of a vector used for the PCA?)**

**Author response:** Thank you for your valuable comment. In PCA analysis, we used **TF-IDF vectors** constructed from the full vocabulary obtained after preprocessing. This resulted in a high-dimensional feature space of **184,412 unique tokens**. PCA was then applied directly to this **TF-IDF matrix** to reduce it to **two principal components.** Furthermore, additional information about the input data and feature representation has already been provided under the **Data Representation** section (lines 142 -149).

**Reviewer 5, Concern # 3. (Proposed hybrid GMM model: is this taking as input only the 2D data from the PCA analysis? Please, clarify, what data are used exactly. On pg. 8 it is stated that the fitness is based on a comparison of predicted to true labels. However, the authors claim that their method is unsupervised. Please, clarify in detail the process. Also, the term 'agent' should be defined clearly)**

**Author response:** We thank the reviewer for this important comment. Basically, we apply PCA to the high-dimensional TF-IDF feature space, reducing it to 2 principal components, and then perform clustering (including our proposed Hybrid GCO-GMM model) on this PCA-reduced data.

This decision was made to significantly reduce computational complexity and improve the efficiency of the optimization and clustering processes. Given the extremely high dimensionality of the TF-IDF matrix (184,412 features), directly applying clustering on the original space would be computationally expensive and prone to over-fitting due to the curse of dimensionality.

Furthermore, although our method is fundamentally unsupervised, we acknowledge the need to clarify the role of true labels. In practice, true labels are not used during the clustering or optimization process. We have refined the fitness evaluation criterion by employing the Silhouette Score a widely accepted internal clustering validation metric. This revision ensures that the optimization process remains entirely unsupervised, aligning with the core principles of clustering-based modeling.

While external metrics such as Accuracy, F-Measure, ARI, and Purity are still depicted after clustering for benchmarking purposes, they are not used during the model optimization process. This revision reflects a more principled and academically consistent approach for evaluating clustering performance in an unsupervised setting.

We have also added a precise description of the term “agent” used in the context of the GCO optimization process. (Please see the highlighted text under the heading “Pseudocode of Proposed Hybrid GMM” (lines 303–312)

**Author Action:** The repository link has been included in the revised manuscript under the "Data and Code Availability" section.

**Reviewer 5, Concern # 4. (4. Why were both the elbow method and the Silhouette score used? Both provide the number of clusters. One of these should be sufficient.)**

**Author response:** Thank you for your valuable feedback. We acknowledge the concern regarding the simultaneous use of both the Elbow Method and the Silhouette Score. Basically, these methods were not used concurrently within the proposed GCO-based hybrid framework, but rather employed for comparative purposes only. Specifically, the Elbow Method and BIC were applied to traditional clustering algorithms (K-means, K-medoids, and standard GMM) to determine baseline cluster numbers, enabling a benchmark comparison against our GCO-optimized model.

The Silhouette Score, on the other hand, is integral to the GCO-based optimization process and serves as the internal clustering evaluation metric guiding the optimization of hyperparameters, including the number of components. Unlike heuristic approaches such as the Elbow Method, the Silhouette Score allows for a more principled, unsupervised optimization strategy.

**Author action:** In the revised manuscript, we have explained this distinction and made explicit the rationale behind including these traditional methods for comparison purposes only, to demonstrate the added value and robustness of our proposed hybrid approach.

The manuscript has also been revised accordingly, as reflected in the main manuscript file highlighted on page 18 of the main manuscript file.

**Reviewer#5, Concern # 5. (On pg.12 reference on the posterior probability, E-step and M-step is made without providing any details of these terms. These should be clarified in the context of this work.)**

**Author response:**

Thank you for pointing this out. Actually we have already defined Expectation-Maximization steps under the Gausssian mixture model heading as highlighted on page 13 line no. 279-290.

**Reviewer#5, Concern #6. (Some benchmark numbers on the computational performance of the hybrid GMM model should be provided, i.e. resources used and computing time for the analysis.)**

**Author response:** Thanks for the comment. The manuscript has also been revised accordingly and included such information under the experimental setup heading in the main manuscript file highlighted on page no. 18 and 21 respectively.

**Reviewer#5, Concern #7. (Comparison of Fig.2 and 4 reveals that the only additional information obtained from the hybrid GMM is the division of the representation in clusters. Is the whole computational effort worth it or could the PCA analysis be used in another more efficient way to reach the detection goal? The authors should clarify this to strengthen their proposed model.)**

**Author response:** Thank you for your perceptive observation regarding the comparative analysis of Figures 2 and 4. The integration of GMM with PCA offers substantial advantages that extend beyond mere visualization. PCA serves as a dimensionality reduction technique, transforming high-dimensional data into a lower-dimensional space while preserving as much variance as possible. However, PCA does not inherently provide clustering capabilities. The incorporation of GMM enables the modeling of data as a mixture of multiple Gaussian distributions, facilitating the identification of underlying data structures and probabilistic cluster assignments.

The manuscript has also been revised accordingly and included such information highlighted in the last paragraph of page no. 23 of the main manuscript file.

**Reviewer#2, Concern # 4. (The clusters in Fig.4 were assigned a label based on the labels in the original data set. How does then the statement of unsupervision hold? Is the whole model applied only to go back to assign labels from the original set? That supports the fact that the labels are needed.**

**Also parts of the data of cluster 1 and 3 are quite spread and include many outliers. How can these be handled in terms of fake news detection?)**

**Author response:** Thank you for your thoughtful comments. We clarify that the hybrid GMM clustering remains unsupervised because the algorithm partitions the data solely based on its internal structure and feature distribution without any label information during the clustering or GCO optimization phases. The subsequent assignment of cluster labels, based on the predominant label in each cluster from the original dataset, is performed only for evaluation purposes using external metrics like Adjusted Rand Index (ARI), and purity and does not influence the unsupervised clustering process itself.

Subsequently, the observation that clusters 1 and 3 exhibit considerable dispersion and contain outliers, we acknowledge that these characteristics can affect the interpretability and robustness of fake news detection. In our work, this dispersion may reflect the inherent variability and complexity of the news data. To address this issue, potential improvements include incorporating outlier detection as a post-processing step using methods such as Isolation Forest or Local Outlier Factor or refining the feature engineering and scaling processes to better accommodate the variability.

**Reviewer#5, Concern # 9. (Fig.5: the elbow method seems not to be efficient for the k-Medoid method, as there are two abrupt changes in the curve.)**

**Author response:** Thank you for your insightful observation regarding Figure 5. We acknowledge that the elbow method exhibits two abrupt changes in the curve when applied to the k-Medoids algorithm, which indicates that this method may not consistently capture the optimal number of clusters in the context of k-Medoids. This behavior could be due to the inherent sensitivity of k-Medoids to initial medoid selection and the discrete, non-smooth nature of its cost function. In our study, the elbow method was used for comparative purposes alongside other evaluation metrics, such as the Silhouette Score, to provide a comprehensive assessment of clustering performance. We agree that alternative or complementary approaches may be more effective for determining the optimal cluster number in k-Medoids, and we plan to explore these strategies in future work.

**Reviewer#5, Concern # 10. (The authors state on pg.15 that "This divergence in the optimal number of clusters across the algorithms highlights the sensitivity of each clustering technique to the underlying data." This would be a very important factor that would hinder the use of the proposed method for the detection of fake news, as the detection would be prone to large errors based on the number of clusters detected. How can the authors overcome this view?)**

**Author response:** Thank you for your thoughtful observation. We appreciate the opportunity to clarify this aspect of our methodology. We acknowledge that the statement on page 15 "This divergence in the optimal number of clusters across the algorithms highlights the sensitivity of each clustering technique to the underlying data" might raise concerns regarding the stability and reliability of the proposed method for fake news detection. Indeed, the sensitivity of clustering algorithms to the number of clusters can impact the consistency of detection outcomes.

To address this concern, we have incorporated the following in our proposed method:

1. Rather than relying on a fixed number of clusters, our approach employs the Group Counseling Optimizer (GCO) to dynamically determine the optimal number of clusters based on the data's intrinsic properties. This adaptive mechanism enhances the model's robustness to variations in data distributions.
2. We utilized a combination of internal clustering validation metrics, such as the Silhouette Score, and external validation measures, including Adjusted Rand Index (ARI) and Purity, to assess clustering quality comprehensively and ensure a more reliable determination of clustering efficacy.
3. To further enhance stability, ensemble clustering methods can be integrated in future.

**Reviewer#5, Concern # 11. (Comparing Fig. 4 and 9: it can be clearly seen that k-means performs the same as the hybrid GMM and should also be computationally faster. How does then the hybrid GMM method perform better, as implied in the manuscript on pg.16and in the conclusions?)**

**Author response:** Thank you for your observation regarding the comparative performance of K-means and the hybrid Gaussian Mixture Model (GMM) as depicted in Figures 4 and 9. While the visual clustering outcomes may appear similar, the underlying performance metrics and methodological approach highlight the superiority of the hybrid GMM in the context of fake news detection.

Table 5 in the main manuscript file demonstrated that the hybrid GMM achieved a Silhouette Score of 0.77, Adjusted Rand Index (ARI) of 0.83, and Purity Score of 0.88, compared to K-means, which recorded a Silhouette Score of 0.65, ARI of 0.72, and Purity Score of 0.79. These improvements indicate that the hybrid GMM more accurately identifies true instances of fake news while minimizing false assignments.

Furthermore, GMM employs probabilistic approach that accounts for the likelihood of data points belonging to each cluster. This soft clustering approach allows for more nuanced distinctions between data points, which is particularly beneficial in the complex landscape of fake news detection.

Additionally, our hybrid GMM approach integrates the Group Counseling Optimizer (GCO) to dynamically determine the optimal number of clusters. This integration enhances the model's adaptability to varying data distributions, further improving its robustness and accuracy in detecting fake news.

While K-means offers computational efficiency due to its simplicity, the additional computational overhead of GMMs is justified by their improved performance and flexibility. The trade-off between computational efficiency and detection accuracy is a critical consideration.

**Reviewer#5, Concern # 8. (Finally, it is not clear how the hybrid GMM model could realistically contribute in fake news detection. First, it does not provide additional valuable information beyond the k-means algorithm. At the same time, the detected clusters need to be labeled to distinguish between 'fake' and 'true' news. In that case, it seems to be practically of higher interest to apply an efficient supervised scheme that can directly classify the news, even if that is computationally more demanding. In that respect, the authors should either strengthen the arguments used and analysis performed or direct their proposed methods to other applications.)**

**Author response:** Thank you for your insightful feedback. While supervised methods are effective when ample labeled data is available, our hybrid GMM addresses scenarios where labeled data is scarce a common challenge in real-world fake news detection.

The hybrid GMM utilizes probabilistic clustering to uncover latent structures in unlabeled data, serving as a preliminary filter to identify potential misinformation. This unsupervised step can be particularly valuable in early detection stages or when new topics emerge rapidly, outpacing the availability of labeled examples.

To enhance practical applicability, in future we plan to integrating semi-supervised learning techniques that combine a small set of labeled data with the unlabeled clusters identified by the GMM.

**Reviewer#5, Minor Comments**

**(a) Some typesetting errors throughout should be corrected, e.g. 'vactorization' on pg.6.**

Thank you for highlighting the typographical error on page 6. We have corrected "vactorization" to "vectorization" and conducted a thorough review of the manuscript to address any additional typesetting issues. We appreciate your attention to detail and commitment to maintaining the quality of the publication.

**(b) Provide references - were missing - for the algorithms used, e.g. GMM (pg.7) or Bayesian Information Criterion (BIC) or k-Medoids (pg.10), when these are first introduced. The same holds for other used concepts/codes/libraries, like scikit (pg.11).**

These references have been added at the respective points in the manuscript where each concept is first introduced. We appreciate your attention to detail and commitment to the rigor of the research.

**(c) Define abbreviations when first used, e.g. PDF on pg.7.**

Thank you for pointing out the omission. We have now defined all abbreviations upon their first occurrence in the manuscript. Specifically, on page 7, "PDF" has been expanded to "Probability Density Function (PDF)" to ensure clarity for all readers.

**(d)** **The title of subsection 2.7 should be changed, as there are no experiments involved.**

Thank you for your update. We have revised the main manuscript file accordingly, changing the heading of subsection 2.7 to “Implementation Configuration.”

**(e) Pg. 15: "We also conducted a comprehensive set of experiments" and "During the experiments", please rephrase as no experiments were performed. The same on all pages referring to 'experiments'.**

We have also reviewed the entire manuscript to identify and amend any other instances where the term "experiments" was inappropriately used, ensuring consistency and accuracy throughout.

We appreciate your attention to detail, which has contributed to enhancing the quality and clarity of our manuscript.

**(f) The legends in Fig.6 are not clear, please define.**

Thank you for your valuable feedback. We have updated the manuscript to clearly define the legends in Fig. 6. Specifically, we now provide detailed explanations of the four covariance types (spherical, tied, diag, and full) used in GMM. These definitions help clarify the structural differences among the models and enhance the interpretability of the BIC-based cluster selection results (Please see highlighted text on page no. 27).

**Reviewer#6, Concern # 1. (The paper introduces a novel hybrid approach combining GMM with GCO for unsupervised fake news detection. This is addresses the limitations of traditional GMM, such as sensitivity to initial parameters and the need for manual cluster selection. However, the paper could benefit from a more detailed discussion on how this hybrid approach compares to other state-of-the-art unsupervised methods in fake news detection.)**

**Author response:** We appreciate the reviewer’s valuable suggestion. To address this concern, we have expanded the discussion section to include a comprehensive comparison between the proposed GMM-GCO hybrid approach and other state-of-the-art unsupervised methods used in fake news detection.

In recent years, various unsupervised techniques have been explored for fake news detection, including K-means, K-medoids, DBSCAN, hierarchical clustering and more recently, deep learning-based clustering frameworks such as autoencoder-based models. These methods have their respective strengths. However, many of these techniques still suffer from challenges like parameter sensitivity, manual selection of the number of clusters, and limited adaptability to high-dimensional textual data.

The proposed GMM-GCO hybrid approach offers several advantages over these traditional and advanced clustering methods. GCO enables automatic tuning of GMM parameters, eliminating the need for manual configuration as seen in traditional GMM, K-means, and hierarchical clustering. It also improves convergence by reducing initialization randomness, leading to more stable and optimal clustering. Empirical results demonstrate that GMM-GCO outperforms K-means, K-medoids, and DBSCAN in terms of clustering accuracy and silhouette scores, indicating better cluster cohesion and separation. Moreover, unlike deep clustering methods that require high computational resources and labeled data, GMM-GCO remains a purely unsupervised and efficient solution, well-suited for real-world fake news detection tasks (Please see highlighted text on page no. 22).

**Reviewer#6, Concern # 2. (The methodology section should provide a more detailed explanation of the GCO algorithm and its integration with the GMM. Specifically, the paper should elaborate on how GCO avoids local optima through its population-based optimization approach and how it enhances GMM's performance in fake news detection by automating parameter tuning and improving convergence to globally optimal solutions.)**

**Author response:** Thank you for the insightful comment. In response, we have revised the Methodology section to provide a more detailed explanation of the Group Counseling Optimization (GCO) algorithm and its integration with the Gaussian Mixture Model (GMM). (Please see highlighted text on page no. 14 under heading no. 2.4).

**Reviewer#6, Concern # 3. (The paper uses Silhouette Score, Adjusted Rand Index, and Purity Score for evaluation. However, the paper should also consider including other metrics like F1-score or precision/recall to provide a more comprehensive evaluation of the model's performance.)**

**Author response:** Thank you for the constructive feedback. In response, we acknowledge the importance of incorporating additional evaluation metrics to provide a more comprehensive assessment of our model's performance.

While the current evaluation focuses on unsupervised clustering metrics Silhouette Score, Adjusted Rand Index (ARI), and Purity Score to assess the cohesion, separation, and agreement with ground truth clusters, we agree that including external validation metrics such as F-measure which is the combination of precision, recall, it can further enrich the analysis, especially when ground truth labels are available. (Please see Table 5 on page no. 30). The addition of these measures strengthens the overall validation of the proposed GMM-GCO approach and confirms its robustness in detecting fake news.

**Reviewer#6, Concern # 4. (The paper should provide more details on the data preprocessing steps, such as how the dataset was balanced and why certain preprocessing techniques were chosen.)**

**Author response:** Thank you for the valuable feedback. In response, we have expanded the Methodology section to include a more detailed explanation of the data preprocessing steps applied before clustering.

The raw dataset underwent several preprocessing stages to ensure data quality, reduce noise, and enhance the effectiveness of the clustering algorithms. (Please see heading no 2.2 and 2.3on page no. 7-14).

**Reviewer#6, Concern # 5. (Ensure all figures, tables, and equations are referenced in the text. For example, Fig. 5 and Fig. 6 are not discussed in detail in the results section.)**

**Author response:** Thank you for pointing that out. We have updated the Results section to ensure that all figures and tables are properly referenced and discussed in the text, including Fig. 5 and Fig. 6.

**Author action:** The manuscript has also been revised accordingly as reflected in the main manuscript file highlighted on page no. 27-28 (line no. 596-608) of the main manuscript file.

**Reviewer#6, Concern # 6. (In Figure 4 and Figure 7 captions lack sufficient detail. For example, Fig. 4 should specify what the clusters represent.)**

**Author response:** Thank you for your valuable feedback. We appreciate the suggestion and have revised the captions for Figure 4 and Figure 7 to provide greater clarity and contextual detail in the main manuscript file (please see lines no. 533 and 623 ).

**Reviewer#6, Concern # 7. (In table 1 should include additional metrics like F1-score or precision/recall for a more comprehensive comparison.)**

**Author response:** Thank you for the constructive feedback. In response, we acknowledge the importance of incorporating additional evaluation metrics to provide a more comprehensive assessment of our model's performance.

While the current evaluation focuses on unsupervised clustering metrics Silhouette Score, Adjusted Rand Index (ARI), and Purity Score to assess the cohesion, separation, and agreement with ground truth clusters, we agree that including external validation metrics such as F-measure which is the combination of precision, recall, it can further enrich the analysis, especially when ground truth labels are available. (Please see Table 5 on page no. 30). The addition of these measures strengthens the overall validation of the proposed GMM-GCO approach and confirms its robustness in detecting fake news.

**Reviewer#6, Concern # 8. (Equation 6, Equation 7 are difficult to follow due to dense notation. Break them down into simpler steps or provide more explanatory text. Ensure all equations are referenced in the text. For example, Equation 10 (fitness function for GCO) is introduced without sufficient context. Explain how this function is used in the optimization process.)**

**Author response:** Thank you for the valuable feedback. To improve clarity, we have revised the description of Equations (6), (7), and (10) into explanatory paragraphs and ensured they are properly referenced in the manuscript. Please see lines no. 271-281 and 338-348 of the main manuscript file respectively.

**Reviewer#6, Concern # 9. (Many references are outdated (from 2017-2019). Include more recent studies (2022-2024) to reflect the latest advancements in fake news detection and unsupervised learning. Some references are incomplete or lack key details. For example, Reference does not include the conference name or page numbers. Ensure all references follow the required citation style. Ensure consistent formatting across references. For example, some references include full first names, while others use initials. Follow the journal's style guide.)**

**Author response:** Thank you for the suggestion. We have updated the references to include recent studies from 2022–2024 and ensured all entries are complete and consistently formatted according to the journal’s style guide.

**Reviewer#7, Concern # 1. ((Comment #3) and Reviewer #2 (Comment #7) suggested the authors to compare their method to some other available methods like the DBScan algorithms. But, the author only provided an empirical discussion about DBScan without showing any solid result ("Our prior experience with DBSCAN yielded unsatisfactory results in terms of clustering accuracy for fake news detection. Although these newer density-based algorithms (QuickDBScan, KDTreeDBSCAN and DenLAC) may offer improvements, the reason to exclude them was likely based on the failure of DBSCAN to meet the requirements for our specific dataset and the added complexity these algorithms could introduce without guaranteed improvements.") I think the authors should further address this comment with some numerical results or real data analysis to show that why DBScan's results are unsatisfactory and the other available methods like QuickDBScan only provide limited improvement. This comment is important to help the readers to understand the advantages of their newly proposed method.)**

**Author response:** Thank you for this important observation. In response to the reviewers’ concerns, we have provided DBSCAN-related results in the supplementary file 1 on page no. 4. These results confirm our earlier observation that DBSCAN achieved significantly lower performance and failed to identify meaningful clusters in the context of fake news detection. This additional analysis clearly demonstrates the performance gap and further justifies our decision to exclude DBSCAN and its variants as baseline methods in the main manuscript.

**Reviewer#7, Minor concerns:**

**Reviewer#7, Concern # 1. (How can users know which cluster is the fake news after applying your method? In your data analysis, you have the ground truth label. So, it's quite easy to find the cluster standing for fake news. But, in reality, people do not have access to such labels. Please discuss how to mitigate this problem.)**

**Author response:** Thank you for highlighting this critical and practical concern. We agree that the absence of ground truth labels in real-world scenarios poses a challenge for directly identifying which cluster represents fake news. However, this limitation is inherent to all unsupervised learning methods, not just our proposed GMM-GCO approach.

Our methodology, while evaluated against labeled data for validation purposes, is specifically designed to be unsupervised and adaptable to real-world settings. The hybrid GMM-GCO model focuses on identifying inherent structures in data based on feature distributions and patterns such as language use, writing style, and semantic inconsistencies that typically differentiate fake news from legitimate content. This clustering can then serve as a foundation for downstream analysis rather than offering a final classification label. To mitigate the lack of labels in practice, we recommend cluster interpretation through feature analysis, where the dominant characteristics of each cluster are examined to infer the likely nature of the content. Furthermore, our method is extendable to semi-supervised setups, where a small, curated set of labeled examples or weak supervision can guide the interpretation of clusters. Unlike deterministic rule-based systems or deep learning approaches requiring extensive labeled datasets, our approach provides a computationally efficient, label-free structure discovery that can be refined iteratively through human-in-the-loop or auxiliary metadata analysis.

Thus, while we acknowledge the limitation of lacking labels in real deployment, our model remains practical by providing a flexible, interpretable foundation that can be aligned with user needs, auxiliary information, or minimal supervision to identify fake news clusters effectively.

**Reviewer#7, Concern # 2 (The authors should double check the mathematical formulas in their manuscript. There are still many typos in the revised version)**

**Author response:** We appreciate the reviewer's observation regarding the need for proofreading and spell-checking.

**Author Action:** To address this, we have thoroughly reviewed the manuscript for grammatical errors, spelling issues, and etc.

**Reviewer#7, Concern # 2.1. ((1) In line 228 and 230 on page 6, descriptive words in formula (2) and (3) should not be italic. But variables like t and n should be italic.)**

**Author response:** Thank you for pointing this out. We have corrected the formatting in formulas (2) and (3) by ensuring that descriptive words remain in regular (non-italic) font, while variables such as t and n are properly italicized, in accordance with mathematical notation standards.

**Reviewer#2, Concern # 2.2. ( In equation (5) on page 7, it should be \Lambda rather than \wedge.)**

**Author response:** Thank you for your observation. We have corrected the notation in Equation (5).

**Reviewer#7, Concern # 2.3. (In line 275 on page 7, "D X D" should be $D \times D$.)**

**Author response:** Thank you for your careful review. The notation has been corrected in line 275 to read as D×D, which properly reflects the dimensional representation in mathematical formatting.

**Reviewer#2, Concern # 2.4. (In equation 7 on page 8, \mathcal{N} is missing before (x|\mu_k,\Sigma_k). The same problem in line 281.)**

**Author response:** Thank you for your observation. We have corrected Equation (7).

**Reviewer#2, Concern # 2.5. In the sentence beginning from line 280, "Whereas \mu_k is the D-dimensional mean vector" should be ", where as \mu_k is the D-dimensional mean vector". Also, there is an unnecessary comma before \Sigma_k. ()**

**Author response:** Thank you for the suggestion. The sentence has been revised for grammatical correctness and clarity.

**Reviewer#2, Concern # 2.6. (In the denominator equation 8, there is an unnecessary word "that".)**

**Author response:** Thank you for your observation. We have corrected Equation (8).

**Reviewer#2, Concern # 2.7. (In the end of line 312, a right parenthesis is missing.)**

**Author response:** Thank you for pointing that out. The sentence has been revised and corrected.
