# Supplementary material for: Unsupervised fake news detection on social media using hybrid Gaussian Mixture Model
Source: PLoS One. 2025 Aug 18;20(8):e0330421. doi: 10.1371/journal.pone.0330421 (PMC12360590; doi:10.1371/journal.pone.0330421)
Supplement: Attachment — Submitted filename: Response-to-Reviewers 19-5.docx [file pone.0330421.s004.docx]

**Original Manuscript ID:** PONE-D-24-50144

**Original Article Title: “**Unsupervised fake news detection on social media using hybrid gaussian mixture model”

**To:** PLOS ONE Editor

**Re:** Response to reviewers

Dear Editor,

Thank you for allowing a submission of our revised manuscript, with an opportunity to address the reviewers’ comments.

We are uploading (a) our point-by-point response to the comments (below) (response to reviewers, under “Response-to-Reviewers Files*”*), (b) an updated manuscript with yellow highlighting indicating changes (as “Revised Article with Changes Highlighted*”*), and (c) a clean updated manuscript without highlights (“Manuscript”*).*

Best regards,

**Reviewer#6, Concern # 1 (The citations in the paper are not presented in sequential order, which disrupts the flow and can confuse readers.).**

**Author response:** Thank you very much for your careful reading and constructive feedback. We sincerely appreciate your attention to detail, which is invaluable in enhancing the overall quality and readability of the manuscript. In response to your observation, we have thoroughly reviewed and reordered all in-text citations to ensure they follow a proper sequential order.

**Reviewer#6, Concern # 2. (Introduction focus on superficial literature review, and near the end, suddenly jumping from the background to explaining that Gaussian mixture models has been widely used modeling dynamic systems without telling what the dynamic system is and how it relates to the previous text.):**

**Author response:** Thank you very much for your valuable and detailed feedback. We appreciate your careful review and acknowledge the importance of maintaining a consistent and accurate reference list. All references were originally generated using Google Scholar to ensure reliable source information; however, we recognize that some entries lacked completeness or formatting consistency. In response, we have meticulously reviewed and revised the entire reference list to correct any missing details and align all entries with the required citation style.

**Reviewer#6, Concern # 3. (The paper presents the core GCO algorithm as a figure, which helps with visualization, but a textual pseudocode version would enhance clarity and reproducibility. Additionally, the algorithm's complexity and convergence behavior should be discussed to provide a deeper understanding of its scalability and efficiency.):**

**Author response:** Thank you very much for your thoughtful and constructive feedback. We appreciate your suggestion to include a textual pseudocode version of the GCO algorithm, which indeed would improve clarity and aid reproducibility (Please see the textual Pseudocode for Hybrid GMM under the heading no. 2. 4 on page no. 14). Accordingly, a detailed discussion of the pseudocode has also been added in the manuscript to complement the existing figure. Furthermore, we have included a discussion on the algorithm’s computational complexity (please see highlighted details on page no. 22 and 23 line no. 500-508)and convergence behavior (please see the highlighted details on page no. 14 ,16 and 17) to better inform readers about its scalability and efficiency. These additions significantly enhance the technical depth and usability of our work.

**Reviewer#6, Concern # 4. (There is a discrepancy between the table reference in the text ("Table 1") and its actual label ("Table 5"). This should be corrected to maintain proper cross-referencing.).**

**Author response:** Thank you very much for your attentive and helpful observation. We sincerely appreciate your thorough review, which has contributed significantly to improve the consistency of our manuscript. The discrepancy between the table reference in the text and its actual label has now been corrected to ensure proper cross-referencing. We have also carefully reviewed all the figures references to avoid similar issues elsewhere in the document.

**Reviewer 6, Concern # 5. (Some sections, like the social media history in the introduction, are too long and could be shortened.)**

**Author response:** Thank you very much for your insightful and constructive feedback. We truly appreciate your careful review and the time you dedicated to improving the quality of our manuscript. In response, we have thoroughly revised the introduction and significantly shortened the section on the history of social media. The updated version now focuses more directly on the context relevant to our research objectives, thereby enhancing clarity and maintaining reader engagement.

**Reviewer 6, Concern # 6. (Equations need consistent formatting, and terms like "Silhouette Score" should be defined before use.)**

**Author response:** Thank you for your valuable feedback. We have revised the manuscript to ensure consistent formatting of all equations throughout the text. Additionally, the term **Silhouette Score** has been defined prior to its first use (Please see the highlighted text under the heading no. 2. 7. 1. 1 on page no. 19 and 20). Specifically, we now introduce it as a clustering evaluation metric that measures how similar an object is to its own cluster compared to other clusters, followed by its mathematical formulation. These changes improve clarity and maintain consistency in technical presentation.

**--------------------------------------------------------------------------------------------------------------------------------**

**Reviewer#7, Concern # 1. (The authors have addressed all my concerns. Yet, I think they should add the response to my comment on "discussions about how users can decide which cluster is the fake news after applying their method" to their discussion part. It will be helpful for users to have some guidance on this point.)**

**Author response:** We sincerely thank the reviewer for the thoughtful feedback and for acknowledging that we have addressed all prior concerns.

We especially appreciate your insightful recommendation to include a discussion in the manuscript on how users can decide which cluster corresponds to fake news after applying the proposed unsupervised method. We agree that this guidance is essential for practical applications, especially in real-world scenarios where ground truth labels may not be available. Furthermore, this concern is indeed valid and reflects a common limitation inherent to all unsupervised learning methods, not just the proposed GMM-GCO hybrid model.

While we utilized a labeled dataset solely for evaluation purposes, our method itself does not rely on these labels during the clustering process. To interpret the resulting clusters post hoc and to evaluate the alignment between discovered clusters and ground truth categories, we applied a majority heuristic labeling strategy. This involved identifying the most frequent ground truth label within each cluster and assigning that label as the representative identity for the cluster. This process enabled us to map the unsupervised clusters to meaningful categories such as mostly true, barely true, false or pants on fire. This mapping was supported by the consistent thematic content observed within each cluster.

For clarity, we have explicitly included this analysis and explanation in the Results and Discussion section of the manuscript (Please see the highlighted text on page no. 25, 26 and 27 line no. 558-606). The section details how the proposed model, when applied to a real-world news dataset, grouped articles into different clusters.

Furthermore, the evaluation metrics Silhouette Score, Adjusted Rand Index and Purity Score presented in Table 1 further validate the effectiveness of our proposed approach compared to traditional clustering techniques like classical GMM, K-means, and K-medoids.
